# Professorial Appointments

**Published:** 1851-07

**Authors:** 


*Professorial Appointments.*—Prof. Paul F. Eve has resigned the chair of Surgery in the Medical Department of the University of Louisville, and is succeeded by Prof. Gross, the former occupant of the chair, lately Prof. of Surgery in the University of the city of New York.

The Editor of the Western Med. and Surg. Journal, in announcing these changes, states, that Prof. Eve was influenced, in resigning his connection with the school, by feelings of personal friendship toward Prof. Gross, learning that the latter had resolved to disolve his connection with the University of New York, and resume his residence at Louisville. This is the more honorable on the part of Prof. Eve, as his course of lectures at Louisville had been remarkaly successful, and, in returning to Augusta, Ga., he does not resume his place in the school with which he was formerly connected. In again entering on the field of his former labors Prof. Gross will doubtless be warmly welcomed by his numerous friends and admirers.

Prof. E. R. Peaslee, of Dartmouth Medical College, N. H., has been appointed to the chair of Physiology, Pathology, and Microscopy in the New York Medical College. Prof. P. is distinguished for his attainments in these branches, and is an experienced teacher. The institution is fortunate in securing his services.

